# The Diary of a "Chronic Appendix"

**Published:** 1914-04-25

**Authors:** 


### THE PATIENTS' COLUMN.

### The Diary of a "Chronic Appendix."

[Again this week we publish one of a series of the less critical "Patients' Point of View" articles. Like the former contribution, the interest is rather in the attitude of mind revealed than in the exercise of the critical faculty.]

First Day.—Entered the ——— Hospital at 11.30 a.m. Endeavoured to adopt matter-of-fact air—failed hopelessly. History recorded in out-patients' department, and conducted to ward. Expected to spend rest of day roaming about—hopes blighted—bathed and put to bed instead. Felt very much out of my element, although Nurse seemed anxious for me to feel "at home." "Lights out" at 7.45 p.m.—earliest time have been to bed for years.

Second Day (Sunday).—Roused at 4 a.m. when just about to get to sleep; horrible night—unearthly noises, apparently coaling warships along the corridor. Arrayed in red coat and sent to chapel. Enjoyed sermon, but some one sang hymns two tones out. Visitors in afternoon—apparently expected to find me half-dead. Very sleepy all day. Glad when "lights out" arrived.

Third Day.—Another horrible night—all the Home Fleet seemed to have "coaled." Found it was only a kitchener being stoked at intervals during night. Ascertained operation takes place in two days. Wish it was over. Read all the papers and half the advertisements. Want to sleep badly.

Fourth Day.—Slightly better night. Night Nurse impressed upon me the desirability of sleep, so accordingly accepted her logic. Mass of literature arrived. Dozed during afternoon—dreamed of most horrible operations—awakened myself by bumping my head on corner of locker.

Operation Day.

Fifth Day (Operation Day).—Decided to keep my "pecker" up. Leave others to judge whether this was so. Arrayed at 2 p.m. in queer garments—looked like a Bashi Bazouk. Gently but firmly led to theatre. Knees none too steady. Theatre looked cold and uninviting, but was warmly welcomed by all. Looked round and found the door shut. Knees continued to be unsteady and incapable of keeping their relative positions. Couldn't see any murderous implements about. Someone gracefully and with much kindness placed a cap over my mouth, and then the local gas-works seemed desirous of emptying its contents into my lungs. Then oblivion. Awoke in bed. Heard my wife's soothing words, but felt totally unable to respond suitably. Realised that the Channel crossing was indeed a rough one. Felt that everyone around me was desirous of my undying gratitude. Shook hands with myself when no one was looking, and shuddered when the thought of the aforesaid gas-works arose in my mind.

Sixth Day.—Felt as "cheap" as the goods in a penny bazaar. Gave everyone a lot of trouble during the night. Felt a beast for doing so, but really couldn't help it. Whatever happened to-day didn't matter to me much.

Seventh Day.—A peculiar kind of day. Couldn't cough, sneeze, or laugh. Have developed a knack of disarranging my bedclothes, as Sister and Nurse seem to be always putting them straight.

Eighth Day.—Visitors in to-day. Could tell from their faces they didn't think much of mine. Felt my chin, and found a beard existing. Rip Van Winkle will have to look out. Fidgety again to-day; bedclothes all out of order. Expect to be told of it if no improvement to-morrow.

Ninth Day (Sunday).—Heard the congregation in chapel singing the hymns. Tried a verse or two with them, and patient in next bed seemed no worse after my vocal efforts. Should dearly love to turn on my side.

Towards Convalescence.

Tenth and Eleventh Days.—Both mainly spent in watching a few solitary flies parade the ceiling.

Twelfth Day.—The delightful performance, consisting of the removal of my "clips," which it was hardly possible for me to enjoy thoroughly, took place at 10 p.m. To my intense relief, the feat of turning on my side was successfully accomplished.

Thirteenth to Eighteenth Days.—Lazily but enjoyably spent in writing, chatting, reading, and witnessing other unfortunates don those queer garments and make their trips to the apartment so intimately connected with the gas-works.

Nineteenth Day.—A real red-letter day. Received, with much delight, the cheerful news of an early return to my home.

Twentieth and Twenty-first Days.—Bedclothes received a severe doing, no doubt due to the excitement aroused by the fact of my approaching discharge. Haven't been cautioned by Sister, so apparently am not the only fidgety patient in the hospital.

"My First Acquaintance with Hospital Life."

Twenty-second Day.—At 10 a.m. donned my usual attire, which seemed to fit where it touched. Carried by two stalwart porters to the hospital entrance, where, after much lifting and hauling, was deposited upon the cushions of a rickety "growler," and reached home safely.

Thus ended my first acquaintance with hospital life, where doctors, sisters, and nurses all combined to make my stay as enjoyable as the circumstances permitted, and it is pleasing to be able to record that their efforts were in every way successful.

